# The Effect of Early Postnatal Nutrition on Human T Cell Leukemia Virus Type 1 Mother-to-Child Transmission: A Systematic Review and Meta-Analysis

**DOI:** 10.3390/v13050819

**Published:** 2021-05-01

**Authors:** Tokuo Miyazawa, Yoshiyuki Hasebe, Masahiko Murase, Motoichiro Sakurai, Kazuo Itabashi, Naohiro Yonemoto

**Affiliations:** 1 Department of Pediatrics, Showa University School of Medicine, Tokyo 142-8666, Japan; yobu_hase@med.showa-u.ac.jp (Y.H.); mush-m@med.showa-u.ac.jp (M.M.); kii0124@yahoo.co.jp (M.S.); kitaba@med.showa-u.ac.jp (K.I.); 2 Aiseikai Memorial Ibaraki Welfare Medical Center, Ibaraki 310-0836, Japan; 3 National Center of Neurology and Psychiatry, Department of Neuropsychopharmacology, National Institute of Mental Health, Tokyo 187-8551, Japan; nyonemoto@gmail.com; 4 Department of Public Health, Juntendo University School of Medicine, Tokyo 113-8421, Japan

**Keywords:** early postnatal nutrition, human T cell leukemia virus, mother-to-child transmission

## Abstract

The main route of mother-to-child transmission (MTCT) of human T cell leukemia virus type 1 is vertical transmission via breastfeeding. Although the most reliable method for preventing MCTC is exclusive formula feeding (ExFF), short-term breastfeeding (STBF) or frozen–thawed breast milk feeding (FTBMF) has been offered as an alternative method if breastfeeding is strongly desired. The aim of this review was to clarify the pooled risk ratio of MCTC of STBF and FTBMF compared with ExFF. This study was registered with PROSPERO (number 42018087317). A literature search of PubMed, CINAHL, the Cochrane Database, EMBASE, and Japanese databases through September 2018 identified 1979 articles, 10 of which met the inclusion criteria. Finally, 11 articles, including these 10 studies and the report of a recent Japanese national cohort study, were included in the meta-analysis. The pooled relative risks of STBF ≤3 months, STBF ≤6 months, and FTBMF compared with ExFF were 0.72 (95% confidence interval (CI): 0.30–1.77; *p* = 0.48), 2.91 (95% CI: 1.69–5.03; *p* = 0.0001), and 1.14 (95% CI: 0.20–6.50; *p* = 0.88), respectively. This meta-analysis showed no statistical difference in the risk of MTCT between STBF ≤3 months and ExFF, but the risk of MTCT significantly increased in STBF ≤6 months.

## 1. Introduction

Human T cell leukemia virus type 1 (HTLV-1) is the first pathogenic retrovirus found in humans. After infection with human T lymphocytes (CD4+), it synthesizes DNA by the action of reverse transcriptase, and exists as a provirus that integrates into the chromosomal DNA of host cells. HTLV-1 carriers are usually asymptomatic, but after a long incubation period, approximately 2% to 7% develop adult T cell leukemia (ATL) [1], and 0.25% to 3.8% develop HTLV-1-associated myelopathy (HAM)/tropical spastic paraparesis (TSP) [2]. A recent meta-analysis shows that HTLV-1 infection is associated with the development of various diseases, other than ATL and HAM/TSP, and people with HTLV-1 infection have a higher risk of death due to any cause than individuals with such an infection [3]. The number of HTLV-1 carriers is estimated to be 5 to 10 million worldwide, and they are unevenly distributed in specific endemic areas, such as Japan (mainly Kyushu and Okinawa regions), West and Central Africa, the Caribbean, and South America [4]. Japan has the largest number of carriers among developed countries, exceeding 1 million [5].

HTLV-1 has weak infectivity, and infection is transmitted by contact between cells via infected lymphocytes. The main transmission routes include mother-to-child transmission (MTCT), sexually transmitted infections, and blood transfusions. In Japan, there is no infection by blood transfusion, because screening tests are conducted, and it is believed that male-to-female transmission by sexual activity accounts for 20% and MTCT accounts for ≥60%, according to a report published in the 1990s [6]. ATL is rarely caused by infection in adulthood, and most ATL cases are derived from MTCT [7]; therefore, the prevention of MTCT is essential.

In recent years, HTLV-1 infection has been spreading to non-endemic areas, such as Tokyo and Osaka. To eradicate HTLV-1-related diseases, such as ATL, the Ministry of Health, Labour, and Welfare of Japan decided to conduct a nationwide HTLV-1 antibody screening of all pregnant women for HTLV-1 antibody by the 30th week of gestation since 2010 [8].

In the 1980s, Japanese research groups reported the presence of HTLV-1-infected cells in the breast milk of carrier mothers [9,10], and subsequent animal studies [11] and epidemiological studies [12,13] have demonstrated that breastfeeding is the main route of MTCT. To prevent transmission via breast milk, the most reliable method is not to feed breast milk containing infected cells, that is, to provide exclusive formula feeding (ExFF). However, ExFF cannot give the mothers and infants the advantage of breastfeeding, such as prevention from other infectious diseases, nutritional effects, economic efficiency, and formation of a good mother–child relationship. Many HTLV-1 carrier mothers are afraid that they cannot form a mother–child attachment because they cannot breastfeed their babies [14]. In recent years, breastfeeding has been actively promoted in Japan. According to a 2015 survey by the Ministry of Health, Labour, and Welfare in Japan, the exclusive breastfeeding rate was 51.3%, and the mixed nutrition rate was 96.5% at the first month of life [15].

As in other countries, the current Japanese manual for the prevention of MTCT by the Health, Labor, and Welfare Science Research Group recommends ExFF for infants born to HTLV-1 carrier mothers [8,16]. If the HTLV-1 carrier mothers strongly desire to breastfeed their babies, short-term breastfeeding (STBF) or frozen–thawed breast milk feeding (FTBMF) has been offered as alternative methods other than ExFF. In the Kagoshima prefecture, which is another endemic area in Japan, >60% of HTLV-1 carrier mothers choose STBF ≤3 months; thus, it is clear that there are many HTLV-1 carrier mothers who desire to breastfeed their babies [17]. However, the evidence for the efficacy of STBF and FTBMF is insufficient, because this is based on small observational studies only.

From April 2012 to December 2015, we prospectively recruited a cohort of HTLV-1 carrier mother at 92 facilities in Japan and calculated the MTCT rates of each feeding option chosen by HTLV-1 carrier mothers [18]. Among the 313 HTLV-1 carrier mothers, 55.0%, 35.1%, 6.1%, and 3.8% selected STBF ≤3 months, ExFF, FTBMF, and long-term breastfeeding, respectively. The MTCT rates of STBF ≤3 months, ExFF, FTBMF, and long-term breastfeeding were 2.3%, 6.4%, 5.3%, and 16.7%, respectively. The risk ratio for STBM compared with ExFF was not statistically different (0.364; 95% confidence interval (CI): 0.116–1.145). Because of the small population, the MTCT rate of FTBMF was not statically reliable.

The aim of this study was to conduct a meta-analysis that combines the recent cohort study we conducted and previous studies to clarify the pooled risk ratio of MTCT of STBF and FTBMF compared with ExFF.

## 2. Material and Methods

This study was registered with PROSPERO (number 42018087317).

### 2.1. Search Strategies

We searched for published studies related to infant feeding and MTCT of HTLV-1 in the following databases from their inception to September 2018: PubMed (from 1949), CINAHL (1981), Cochrane Databases (from 1939), and EMBASE (from 1947). The search strategy included the terms below: (“HTLV” or “human T-lymphotropic” or “human T cell leukemia”) and ((“mother” and “child”) or (“milk” or “vertical”)) and (“transmission” or “infection).

We also searched the following Japanese databases: ICHUSHI (from 1983), CiNii (from 1881), KAKEN, and the Database of Health Labour Science Research Grant. ICHUSHI contains bibliographic citations and abstracts from biomedical journals and other serial publications published in Japan. CiNii contains information on academic articles published in academic society journals, university research bulletins, or articles included in the National Diet Library’s Japanese Periodical Index Database. KAKEN is a public database that includes information on adopted projects, assessment, and research achievements from the Grants-in-Aid for Scientific Research (KAKENHI) Program. Since these four databases are electronic databases in Japanese, we used comparable Japanese terms. We also examined the list of references in the included studies.

### 2.2. Definition of Terms

The definition of STBF varies among articles. In this study, we defined STBF as breastfeeding within three months of age (STBF ≤3 months) or within six months of age (STBF ≤6 months). MTCT was confirmed by the detection of the HTLV-1 antibody in infants ≥12 months.

### 2.3. Inclusion Criteria

We included studies if they met the following criteria: the mothers were found to be an HTLV-1 carrier by an antibody test; the HTLV-1 antibody tests were performed on their children aged >12 months to <15 years; and the MTCT rate of the intervention group (STBF or FTBMF) was compared with that of the ExFF group (control).

### 2.4. Exclusion Criteria

We excluded studies if the study population included children aged <12 months or >15 years, who had a history of blood transfusion, had married, or had sexual intercourse.

### 2.5. Extraction of Data

The titles and abstracts of the studies were retrieved using the search strategy, and those from additional sources were screened independently by two of the four reviewers (T.M., H.Y., M.M., and M.S.) to identify studies that potentially met the inclusion criteria. Second, the full text of these potentially eligible studies was retrieved and independently assessed for eligibility by two reviewers. Any disagreement between the two reviewers regarding the eligibility of a particular study was resolved through discussion with a third reviewer.

### 2.6. Quality Assessment

To assess the quality of the included studies, the Newcastle–Ottawa Scale (NOS) was applied in this review [19]. According to this scale, each study is evaluated according to eight items, categorized into three sections: selection, comparability, and exposure. The total score ranges from 0 to 9, and studies with scores ≥5 are generally considered as having a high enough quality to be included in the meta-analysis. Each study was independently assessed by two authors (T.M. and K.I.). If the evaluation of the two did not match, the third author was involved with the final decision.

### 2.7. Data Synthesis and Statistical Analysis

Our primary outcome was MTCT transmission rate by feeding strategy. We provided a synthesis of the findings from the included studies, structured around the type of intervention, study population characteristics, and type of outcome. In addition, we provided summaries of the intervention effects for each study by calculating the risk ratio.

The meta-analysis was carried out using Review Manager version 5.3. (The Nordic Cochrane Centre, The Cochrane Collaboration, Copenhagen, Denmark, 2014). The pooled relative risks (RRs) and 95% CIs were calculated using the Mantel–Haenszel random effect model. Heterogeneity across the studies was assessed using the *I*^2^ statistic, and the *I*^2^ values were considered low, moderate, and high, with upper limits of 25%, 50%, and 75%, respectively [20]. Publication bias was assessed using a funnel plot. Statistical significance was set at *p* < 0.05.

## 3. Results

### 3.1. Search and Article Selection

**Figure 1:**
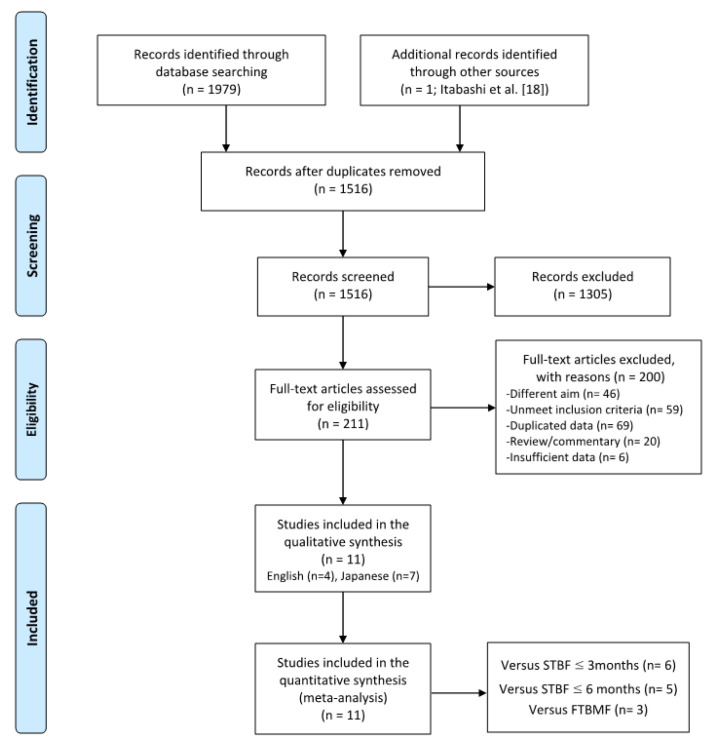
Flow diagram of the study selection. STBF, short-term breastfeeding; FTBMF, frozen–thawed breast milk feeding.

**Figure 2:**
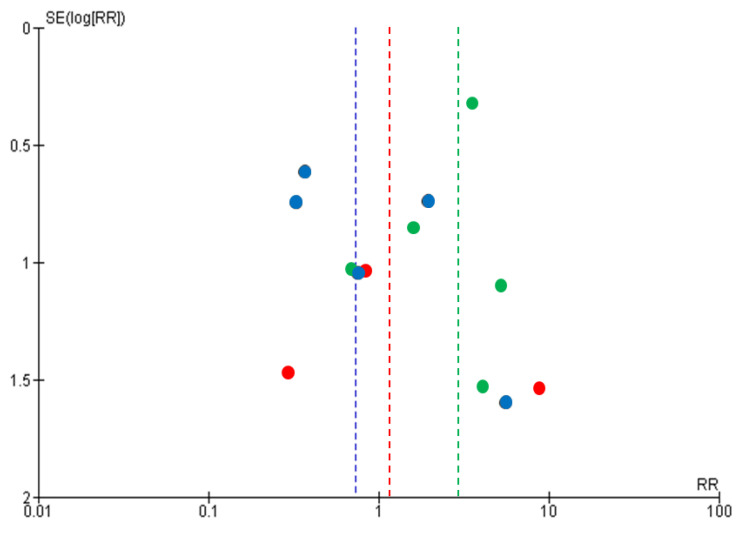
Funnel plots of the included studies. Blue indicates the STBF ≤3 months compared with ExFF, green indicates the STBF ≤6 months compared with ExFF, and red indicates the FTBMF compared with ExFF.

The search strategy identified 1797 records for potential inclusion in the study: PubMed (n = 330), CINAHL (n = 18), EMBASE (n = 589), ICHUSHI (n = 788), CiNii (n = 28), KAKEN (n = 1), and the Database of Health Labour Sciences Research Grant (n = 43) (Figure 1). After reviewing the title and abstract of 1798 articles, including the report of our recent cohort study [18], the full text of 211 articles was obtained for further assessment of their eligibility. Assessment of the full-text articles yielded 11 articles, and these 11 articles were included in the meta-analysis. The characteristics of the included studies that compared the MTCT rates between ExFF and STBF ≤3 months, STBF ≤6 months, and FTBMF are shown in Table 1, Table 2 and Table 3, respectively. According to the NOS, seven of the 11 articles achieved a score ≥5 (see Table 4). Figure 2 shows the funnel plot of the adopted articles in this meta-analysis. Since the number of articles included in each comparison is small, it is difficult to evaluate publication bias, although the distribution seems to be symmetric.

**Selection (a maximum of one star can be given for each numbered item)**

1. Representativeness of the exposed cohort
  - Truly representative of the average HTLV-1 carrier mothers and their children in the community ★
  - Somewhat representative of the average HTLV-1 carrier mothers and their children in the community ★
  - Selected group of users e.g., nurses, volunteers
  - No description of the derivation of the cohort
2. Selection of the non-exposed cohort
  - Drawn from the same community as the exposed cohort ★
  - Drawn from a different source
  - No description of the derivation of the non-exposed cohort
3. Ascertainment of exposure
  - Secure record (e.g., surgical records) ★
  - Structured interview ★
  - Written self-report
  - No description
4. Demonstration that outcome of interest was not present at start of study
  - Yes ★
  - No

**Comparability (a maximum of two stars can be given)**

1. Comparability of cohorts on the basis of the design or analysis
  - Study controls for maternal anti-HTLV-1 antibody titer and/or maternal HTLV-1 pro-viral load ★
  - Study controls for any additional factor ★

**Outcome (a maximum of one star can be given for each numbered item)**

1. Assessment of outcome
  - Independent blind assessment ★
  - Record linkage ★
  - Self-report
  - No description
2. Was follow-up long enough for outcomes to occur
  - Yes ★
  - No
3. Adequacy of follow-up of cohorts
  - Complete follow-up ★
  - Subjects lost to follow-up unlikely introduce bias, with follow-up >70% or description provided of those lost ★
  - Follow-up rate <70% and no description of those lost
  - No statement

### 3.2. Comparison between STBF ≤3 Months and ExFF

**Figure 3:**
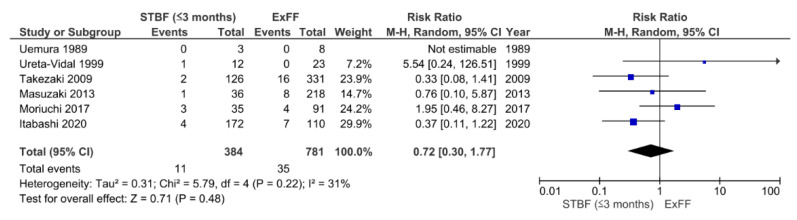
Forest plots of the RRs of mother-to-child transmission of human T cell leukemia virus type 1 in the STBF ≤3 months group compared with the ExFF group. STBF, short-term breastfeeding; ExFF, exclusive formula feeding; M-H, Mantel–Haenszel; CI, confidence interval; RR, relative risk.

We identified a total of five retrospective studies [21,22,23,24,25] and one prospective [18] study that were eligible for the comparison of STBF ≤3 months and ExFF (Figure 3). There were 11 cases of HTLV-1 positivity among 384 children in the STBF ≤3 months group compared with 35 cases among 781 children in the ExFF group (pooled RR: 0.72; 95% CI: 0.30–1.77; *p* = 0.48). There was moderate heterogeneity between the studies (*I*^2^ = 31%, *p* = 0.22).

### 3.3. Comparison between STBF ≤6 Months and ExFF

**Figure 4:**
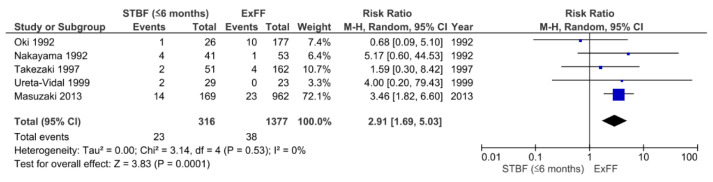
Forest plots of the RRs of mother-to-child transmission of human T cell leukemia virus type 1 in the STBF ≤6 months group compared with the ExFF group. STBF, short-term breastfeeding; ExFF, exclusive formula feeding; M-H, Mantel–Haenszel; CI, confidence interval; RR, relative risk.

We identified a total of four retrospective studies [22,24,26,27] and one prospective study [28] that were eligible for the comparison of STBF ≤6 months and ExFF (Figure 4). There were 23 cases of HTLV-1 positivity among 316 children in the STBF ≤6 months group compared with 38 cases among 1377 participants in the ExFF group (pooled RR: 2.91; 95% CI: 1.69–5.03; *p* = 0.0001). There was low heterogeneity between the studies (*I*^2^ = 0%, *p* = 0.53).

### 3.4. Comparison between FTBMF and ExFF

**Figure 5:**
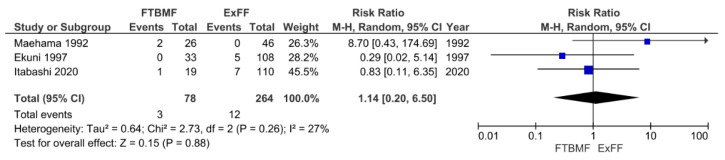
Forest plots of the RRs of mother-to-child transmission of human T cell leukemia virus type 1 in the FTBMF group compared with the ExFF group. FTBMF, frozen–thawed breast milk feeding; ExFF, exclusive formula feeding; M-H, Mantel–Haenszel; CI, confidence interval; RR, risk ratio.

We identified only three prospective studies [18,29,30] that were eligible for the comparison of FTBMF and ExFF (Figure 5). There were three cases of HTLV-1 positivity among 78 children in the FTBMF group compared with 12 cases among 264 children in the ExFF group (pooled RRL: 1.14; 95% CI: 0.20–6.50; *p* = 0.88). In the study of Maehama et al. [29], the duration of FTBMF was limited to one month, followed by formula feeding. The study of Ekuni [30] did not describe the duration of FTBMF, but the follow-up study by the same authors [31] stated that the mean duration of FTBMF was two months (varying from two weeks to six months). There was moderate heterogeneity between the studies (*I*^2^ = 27%, *p* = 0.26).

## 4. Discussion

In the recent technical report on HTLV-1, the World Health Organization recommends that “available data should be further analyzed to better define the risk of HTLV-1 transmission associated with specific durations of breastfeeding, balanced with the risks of other adverse health outcomes that may result from reduced breastfeeding” [32]. This meta-analysis combined the data of a recent cohort study we conducted and those of previous studies to clarify the pooled risk ratio of MTCT of STBF and FTBMF compared with ExFF, and the results showed that the risk of MTCT between STBF ≤3 months and ExFF had no statistical difference, but it significantly increased at STBF ≤6 months.

The preventive effect of short-term breastfeeding on MTCT is assumed to be due to the suppression of infection by the neutralizing antibody that is transplacentally transferred from the mother in utero [33]. STBF <3 or <6 months has been reported to have protective effects in endemic areas in Japan, but these are based on small studies, and no evidence has been established at this time.

The duration of breastfeeding is considered to be one of the factors affecting the MTCT rate, but there are no studies directly comparing the MTCT rate with breastfeeding periods <3 and <6 months. As a result of this meta-analysis, there was no statistical difference in the risk ratio for MTCT of STBF ≤3 months compared with ExFF; however, the number of subjects and events was small. Meanwhile, the risk of MTCT for STBF ≤6 months was 2.91 times higher than that for ExFF. This result suggests that STBF ≤3 months may be as effective as ExFF in preventing MTCT, but continued breastfeeding >3 months may increase the risk of MTCT.

In our recent cohort study, approximately 8% of the mothers who selected STBF ≤3 months were unable to wean breastfeeding by six months of life [18]. If the mother selects STBF, it is necessary to explain in advance that it is not easy to stop breastfeeding by three months and to support weaning from approximately two months of life.

High maternal HTLV-1 pro-viral load (PVL) has been reported to be a risk factor of MTCT [22,34,35]. Among the studies examined in this meta-analysis, only the study of Ureta-Vidal et al. [22] showed that high PVL increases the risk of MTCT by logistic analysis adjusted for breastfeeding duration. Further studies are needed to determine whether STBF >3 months increases the risk of MTCT, even when maternal PVL is low.

It is well-known that antiretroviral therapy (ART) can reduce the risk of MTCT in patients with human immunodeficiency virus, and similar effects are expected [36]. Clinical studies are needed on the efficacy and safety of ART on breastfeeding HTLV-1 mothers, as it will allow mothers to be offered with alternative feeding options.

A meta-analysis published in 2018 by Boostani et al. [37] showed that STBF ≤6 months does not increase the risk of MTCT compared with ExFF, which is different from our analysis.

The reason for this difference is unknown, but while Boostani et al. [37] extracted only articles written in English, we included articles written in Japanese, large public research reports that have not been published, and the results of the latest nationwide cohort study in Japan. Some of the articles included by Boostani et al. [37] overlap with the Japanese articles and public reports; thus, we adopted the one with the highest cumulative number of cases.

FTBMF has been reported to be effective in preventing MTCT, because infected T lymphocytes are destroyed by freezing and thawing treatment, and the infectivity is inactivated [30]. In this meta-analysis, there were no differences in the MTCT rate between FTBMF and ExFF, but the number of subjects analyzed was small, and evidence on its preventive effect was insufficient. Moreover, due to the short duration of FTBMF in the adopted studies, the effect may be due to STBF. In fact, FTBMF is a time-consuming activity, and it might be difficult for mothers to continue FTBMF in the long term.

This study has several limitations. First, most of the studies included in the meta-analysis are retrospective observational studies, and there are no randomized controlled trials. These retrospective studies have low follow-up rates, and the details of the nutritional methods may be inaccurate. In addition, almost none of the studies described the proportion of mixed nutrition or the actual period of breastfeeding in the STBF group. Second, there is an inconsistent timing of antibody testing in children. Seroconversion of the HTLV-1 antibody is thought to occur by the age of three years [38,39]. The Japanese manual recommends antibody testing for children aged three years [16]. In most of the studies adopted in this meta-analysis, the antibody tests were performed at the age of one year to two years; thus, the MTCT rate may have been underestimated. Third, all of the studies adopted in the meta-analysis were conducted in Japan, except for a report from French Guyana; thus, it is unclear whether our results are applicable to individuals from countries other than Japan. Further research is needed in various countries with different settings.

## 5. Conclusions

This meta-analysis showed that there was no statistical difference in the risk of MTCT between STBF ≤3 months and ExFF, but the risk of MTCT significantly increased in STBF ≤6 months. Although the superiority of ExFF remains unchanged in the prevention of MTCT, it is necessary to understand the risks of prolonged breastfeeding when STBF was selected and to establish a system to support mothers and children.

## Figures and Tables

**Table 1:** Characteristics of the included studies comparing the MTCT rates between ExFF and STBF ≤3 months.

Author(s), Year	Study Area	Study Period	Study Population	ExFF	STBF		Timing of Antibody Test of Children	Study Design	Reference Number
				Seroconversion n/N (%)	Definition	Seroconversion n/N (%)			
Uemura et al., 1989	Okayama, Japan	NA	Children born to HTLV-1 carrier mothers, including older siblings	0/8 (0%)	Breastfeeding <3 months	0/3 (0%)	≥12 months	Retrospective	[21]
Ureta-Vidal et al., 1999	French Guyana	1989 t–NA	Children born to HTLV-1 carrier mothers, including older siblings	0/23 (0%)	Breastfeeding ≤3 months	1/12 (8.3%)	18 months to 12 years	Retrospective	[22]
Takezaki, 2009	Kagoshima, Japan	1986–2006	Children born to HTLV-1 carrier mothers	16/331 (4.8%)	Breastfeeding <3 months	2/126 (1.6%)	≥18 months	Retrospective	[23]
Masuzaki et al., 2013	Nagasaki, Japan	1998–2008	Children born to HTLV-1 carrier mothers	8/218 (3.7%)	Breastfeeding <3 months	1/36 (3.7%)	Three years	Retrospective	[24]
Moriuchi et al., 2017	Nagasaki, Japan	2011–2017	Children born to HTLV-1 carrier mothers	4/91 (4.4%)	Breastfeeding ≤90 days	3/35 (8.5%)	≥36 months	Retrospective	[25]
Itabashi et al., 2020	Japan (national survey)	2012–2015	Children born to HTLV-1 carrier mothers	7/110 (6.4%)	Breastfeeding ≤3 months	4/172 (2.3%)	Three years	Prospective	[18]
Total				35/781 (4.5%)		11/384 (2.9%)			

MTCT, mother-to-child transmission; ExFF, exclusive formula feeding; STBF, short-term breastfeeding; NA, not assessed; HTLV-1, human T cell leukemia virus type 1.

**Table 2:** Characteristics of included studies comparing MTCT rates between ExFF and STBF ≤6 months.

Author(s), Year	Study Area	Study Period	Study Population	ExFF	STBF		Timing of Antibody Test of Children	Study Design	Reference Number
				Seroconversion n/N (%)	Definition	Seroconversion n/N (%)			
Nakayama et al., 1992	Kagoshima, Japan (single-center survey)	1986–1990	ExFF: children born to HTLV-1 carrier mothers STBF: older siblings	1/53 (1.9%)	Breastfeeding ≤6 months	4/41 (9.8%)	1–5 years	Retrospective	[26]
Oki et al., 1992	Kagoshima and Miyazaki, Japan	1986–1991	Children born to HTLV-1 carrier mothers	10/177 (5.6%)	Breastfeeding ≤6 months	1/26 (3.8%)	1–3 years	Prospective	[27]
Takezaki et al., 1997	Tsusima and Kamigoto, Nagasaki, Japan	1985–1991	Children born to HTLV-1 carrier mothers	4/162 (2.5%)	Breastfeeding ≤6 months	2/51 (3.9%)	≥30 months	Retrospective	[28]
Ureta-Vidal et al., 1999	French Guyana	1989–NA	Children born to HTLV-1 carrier mothers, including older siblings	0/23 (0%)	Breastfeeding ≤6 months	2/29 (6.9%)	18 months to 12 years	Retrospective	[22]
Masuzaki et al., 2013	Nagasaki, Japan	1987–1997	Children born to HTLV-1 carrier mothers	23/962 (2.4%)	Breastfeeding <6 months	14/169 (8.3%)	Three years	Retrospective	[24]
Total				38/1377 (2.8%)		23/316 (7.3%)			

MTCT, mother-to-child transmission; ExFF, exclusive formula feeding; STBF, short-term breastfeeding; NA, not assessed; HTLV-1, human T cell leukemia virus type 1.

**Table 3:** Characteristics of included studies comparing MTCT rates between ExFF and FTBMT.

Author, Year	Study Area	Study Period	Study Population	ExFF	FTBMF		Timing of Antibody Test of Children	Study Design	Reference Number
				Seroconversion n/N (%)	Definition	Seroconversion n/N (%)			
Maehama et al., 1992	Okinawa, Japan	1986–1989	Children born to HTLV-1 carrier mothers	0/46 (0%)	12 h of freezing at a home freezer followed by natural thawing	2/26 (7.7%)	1–3 years	Prospective	[29]
Ekuni, 1997	Okinawa, Japan	1983–1984	Children born to HTLV-1 carrier mothers	5/108 (4.6%)	12 h of freezing at −20 °C followed by natural thawing	0/33 (0%)	24 months	Prospective	[30]
Itabashi et al., 2020	Japan (national survey)	2012–2015	Children born to HTLV-1 carrier mothers	7/110 (6.4%)	24 h of freezing in a home freezer followed by natural thawing	1/19 (5.3%)	Three years	Prospective	[18]
Total				12/264 (4.5%)		3/78 (3.8%)			

MTCT, mother-to-child transmission; ExFF, exclusive formula feeding; FTBMF, frozen–thawed breast milk feeding; STBF, short-term breastfeeding; HTLV-1, human T cell leukemia virus type 1.

**Table 4:** Quality assessment of the studies by the NOS.

Author, Year	Selection				Comparability	Outcome			Total Quality Score	Reference Number	Language	Types of Article
	Representativeness of Exposed Cohort	Selection of the Non-Exposed Cohort	Ascertainment of Exposure	Demonstration That Outcome of Interest was not Present at Start of Study	Comparability of Cohorts on the Basis of the Design or Analysis	Assessment of Outcome	Follow-Up Length for Outcomes to Occur	Adequacy of Follow-Up				
Uemura et al., 1989	★			★		★		★	4	[21]	Japanese	Meeting abstract
Nakayama et al., 1992	★			★		★			3	[26]	Japanese	Original article
Oki et al., 1992	★	★		★		★	★		5	[28]	English	Original article
Maehama et al., 1992			★	★		★	★		4	[29]	Japanese	Original article
Takezaki et al., 1997	★	★	★	★		★	★		6	[27]	English	Original article
Ekuni, 1997	★	★		★		★			4	[30]	Japanese	Original article
Ureta-Vidal et al., 1999	★	★		★	★★	★	★	★	8	[22]	English	Original article
Takezaki, 2009	★	★		★		★	★		5	[23]	Japanese	Public research report
Masuzaki et al., 2013	★	★		★		★	★		5	[24]	Japanese	Public research report
Moriuchi et al., 2017	★	★		★		★	★		5	[25]	Japanese	Public research report
Itabashi et al., 2020	★	★	★	★		★	★		6	[18]	English	Original article

NOS, Newcastle–Ottawa Scale.

## Data Availability

The datasets used in this study can be found in the full-text articles used in this systematic review.
